# Late Donor-Site Complications Following Free Fibular Flap Harvest: A Report of a Rare Case

**DOI:** 10.7759/cureus.68177

**Published:** 2024-08-30

**Authors:** Dinah F Alahmadi, Ibrahim R Halawani, Abdulaziz M Alghamdi, Mohammed H Shawosh, Adil N Althobaity

**Affiliations:** 1 Medicine, King Saud bin Abdulaziz University for Health Sciences, Jeddah, SAU; 2 Medicine, King Abdulaziz University, Jeddah, SAU; 3 Plastic Surgery, King Fahad Armed Forces Hospital, Jeddah, SAU; 4 Plastic and Reconstructive Surgery, King Abdulaziz Medical City, Jeddah National Hospital, Jeddah, SAU

**Keywords:** fibular flap complication, compartment syndrome, donor-site morbidity, delayed hematoma, fibular flap harvest

## Abstract

Mandibulectomy with free fibular flap reconstruction is a well-established procedure in head and neck surgery, recognized for its functional and aesthetic outcomes. However, this procedure carries certain risks, including the rare occurrence of late-onset donor site morbidities such as compartment syndrome. We report the case of a 67-year-old male with multiple comorbidities who developed compartment syndrome due to delayed hematoma formation at the donor site after undergoing segmental mandibulectomy with left fibular osteocutaneous free flap reconstruction. The patient, who was on antithrombotic therapy for ischemic heart disease, presented with severe calf pain and swelling on the 11th postoperative day, necessitating urgent surgical intervention. Hematoma evacuation and meticulous hemostasis led to a successful outcome. This case underscores the importance of thorough preoperative evaluation, precise surgical technique, and vigilant postoperative monitoring, especially in patients with significant comorbidities. Prompt recognition and management of delayed hematoma are essential to prevent further complications. Enhanced awareness and early intervention are critical in addressing this rare complication, and further research is warranted to establish standardized guidelines and identify specific risk factors for delayed hematoma formation.

## Introduction

Mandibulectomy with free fibular flap reconstruction is a well-established procedure in head and neck reconstruction known for delivering remarkable functional and aesthetic outcomes in patients undergoing extensive mandibular resections. Despite its effectiveness, the procedure carries certain risks and complications. While immediate postoperative complications are well documented and typically managed, late-onset donor site morbidities, such as hematoma formation, present a unique challenge due to their rarity and potential severity.

Hematoma formation at the donor site following free fibular flap harvest is uncommon and generally considered an early complication [1]. However, cases of delayed hematoma formation, appearing days to weeks postoperatively, have been sporadically reported in the literature, although often with limited detail. This delayed presentation poses a diagnostic challenge and requires prompt recognition and intervention to prevent further complications, including infection, necrosis, or impaired wound healing.

To date, only four cases of acute compartment syndrome following fibular flap harvest have been reported in the literature, with no documented instances of compartment syndrome arising from hematoma formation [2].

In this report, we present the case of a 67-year-old male who developed symptoms of compartment syndrome secondary to a late-onset hematoma at the donor site following fibular flap harvest for mandibular reconstruction. This case aims to highlight the clinical presentation, management strategies, and outcomes associated with this rare complication. Additionally, we discuss potential risk factors for delayed hematoma formation and underscore the importance of vigilant postoperative monitoring and early intervention in such cases.

This case underscores the necessity of thorough preoperative evaluation, meticulous surgical technique, and vigilant postoperative care to minimize the risk of complications associated with this complex surgical procedure.

## Case presentation

A 67-year-old male, an ex-heavy smoker with multiple comorbidities including type 2 diabetes mellitus, hypertension, dyslipidemia, ischemic heart disease, and hypothyroidism, presented with a long-standing oral cavity lesion first noted two years prior. The lesion was initially painless but had occasionally bled over time. The patient had no history of malignancies or trauma but had undergone coronary artery bypass surgery nearly 10 years ago. His medications at presentation included Plavix 75 mg and aspirin 100 mg, in addition to antihypertensives and antidiabetics.

Head and neck examination revealed palpable left cervical lymph nodes, and oral cavity examination showed a hard, non-ulcerating, non-bleeding mixed white-reddish lesion involving the 45-47 teeth region. Other physical examinations were unremarkable. A biopsy of the lesion confirmed a well-differentiated squamous cell carcinoma of the gingiva.

A multidisciplinary team, including head and neck, plastic, and oral and maxillofacial surgeons, was assembled to perform a bilateral neck dissection, right mandibulectomy with tumor excision, and right mandible reconstruction using a left non-innervated osteocutaneous free fibula flap. The patient consented to the management plan and was optimized by the anesthesia team, who discontinued his antithrombotic medications.

The patient was taken to the operating room under general anesthesia. The head and neck surgery team performed the bilateral neck dissection and right mandibulectomy with complete tumor excision. The maxillofacial team measured and prepared the plates for bony fixation, pre-drilling holes, and bending plates as needed for reconstruction.

Simultaneously, the plastic surgery team harvested the left osteocutaneous free fibula flap. A 4 × 6 cm skin paddle was designed, and a tourniquet was applied to the thigh, elevated to 300 mmHg. Dissection proceeded through the anterior harvesting approach, down to the anterior intermuscular septum, which was incised and opened. The muscles were elevated, and the interosseous membrane was identified, incised, and opened.

A posterior skin incision was made, and dissection continued to the muscle fascia, which was then incised. The skin paddle was completely elevated off the soleus muscle, and the transverse intermuscular septum was identified, incised, and opened. Proximal and distal osteotomies of the fibula were performed with an oscillating saw. The pedicle was separated, the tourniquet was released, and the flap was allowed to perfuse before final harvesting.

The plastic surgery and maxillofacial surgery teams completed flap fixation and microvascular anastomosis at the recipient site. At the donor site, compartments were left open, and the subcutaneous tissue and skin were primarily sutured without fenestrated skin grafts. The surgery proceeded without complications, and the patient was transferred to the intensive care unit for close monitoring of flap viability and potential complications.

**Figure 1:**
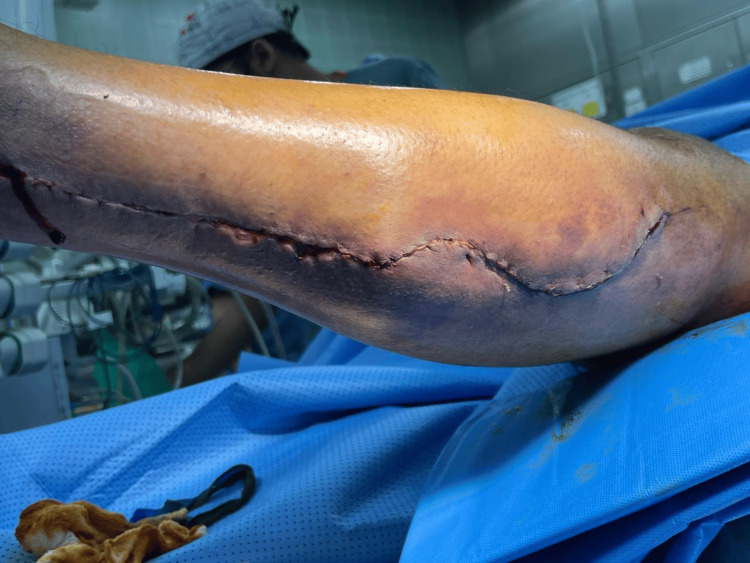
Preoperative view demonstrating swelling, ecchymosis, and discharge from the wound site

A few days later, the patient was moved to the ward and resumed his previous doses of aspirin and clopidogrel based on cardiology recommendations. On the 11th postoperative day, he developed severe calf pain after attempting to stand for the first time since surgery. Examination revealed a swollen, tense, and shiny leg with non-palpable peripheral pulses, although they were detected by Doppler ultrasound. Sensation at the foot was diminished across all dermatomes, and he experienced extreme pain upon passive plantarflexion and dorsiflexion. These symptoms raised concerns for acute compartment syndrome, prompting urgent exploration of the donor site (Figure 1).

**Figure 2:**
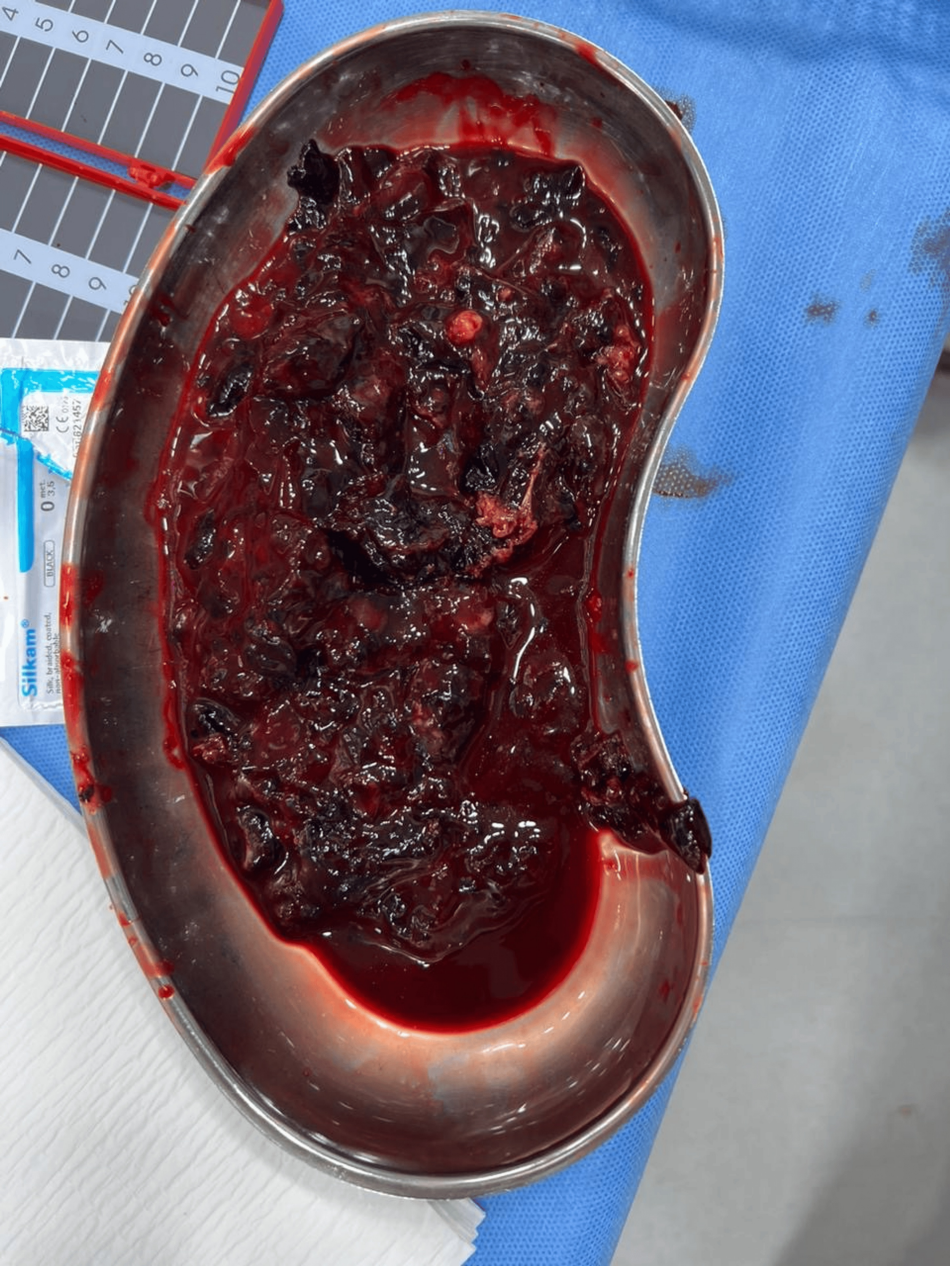
Hematoma evacuated from the leg compartments

Upon incising the healed skin and subcutaneous tissue, approximately 200 cc of clotted hematoma was immediately evacuated from both the superficial and deep posterior compartments (Figure 2). After the complete evacuation of the hematoma, the leg was irrigated with three liters of normal saline, and small bleeding vessels were cauterized using monopolar electrocautery. Following control of active bleeding and oozing, additional irrigation with another three liters of warm normal saline was performed. The muscles and deep dermal layers were loosely approximated with sutures and staples. The donor site of the previously harvested skin paddle was left open, and vacuum-assisted closure (VAC) sponge was applied with continuous suction at 80 mmHg. The patient tolerated the procedure well and was transferred to the recovery room in stable condition.

All symptoms improved following the hematoma evacuation, and the dual antithrombotic therapy was temporarily discontinued for 12 days. The patient recovered well and was discharged in good condition after the VAC sponge was removed on the 20th day post-evacuation. Two months later, follow-up revealed that the left leg wound had healed without further complications.

## Discussion

Overview of the case

This case involves a 67-year-old patient who developed compartment syndrome due to a delayed hematoma at the donor site following segmental mandibulectomy with left fibular osteocutaneous free flap reconstruction. The patient’s extensive medical history, including type 2 diabetes mellitus, hypertension, dyslipidemia, ischemic heart disease, hypothyroidism, and long-term antithrombotic therapy, posed significant surgical and postoperative challenges. The rarity of delayed hematoma formation, typically an early postoperative complication, highlights the clinical significance of this case and underscores the importance of prompt recognition and management.

Hematoma formation following free fibular flap harvesting is generally considered an early postoperative complication, with most cases occurring within the first few days after surgery. Li et al. conducted a study assessing the risks of both early and late donor site morbidities. Although the study did not primarily focus on hematoma formation, they reported an early postoperative hematoma in one patient, with no late complications involving hematoma formation [3].

Similarly, a study by Ali et al. found that neither early nor late complications included hematoma formation, with most complications involving graft loss, gait disturbances, ankle instability, sensory loss, and deformities. This further emphasizes the rarity of postoperative hematoma formation and the need for its consideration in cases similar to ours [4].

Surgical techniques play a critical role in minimizing complications. Graham et al., as discussed in a study by Ling et al., highlighted the advantages of muscle-sparing flap elevation. This approach reduces dead space at the donor site, thereby decreasing the likelihood of blood pooling and hematoma formation. Additionally, it strengthens the donor leg postoperatively by preserving more muscle, simplifies the placement of the skin flap over the fibula, and allows for safer, more precise dissection of blood vessels. Implementing such advanced techniques can significantly reduce the risk of hematoma formation, particularly in patients with multiple comorbidities and those on antithrombotic medication, ultimately enhancing overall surgical outcomes [1,5].

Potential risk factors

A wide array of factors contributed to the formation of a delayed hematoma in our patient. His medical comorbidities, including type 2 diabetes mellitus, hypertension, ischemic heart disease, dyslipidemia, and hypothyroidism, not only delayed wound healing but also increased the risk of bleeding. This is consistent with findings from both Ali et al. and Medina et al., who reported that patients with such comorbidities are at a higher risk of developing complications, as these conditions are known to complicate outcomes and delay recovery [4,6].

Furthermore, the reintroduction of the patient’s antithrombotic medication postoperatively likely contributed to hematoma formation, despite the preoperative discontinuation of the medication. Jakobsson et al. also found that hematoma formation was commonly associated with the use of antithrombotic medication, underscoring the challenges of managing patients with these therapies [7].

Additional factors suggested in the literature, such as the application of a tourniquet, blood vessel handling, and suturing techniques, may also play a role in the formation of a delayed hematoma. Ensuring meticulous hemostasis and proper surgical wound management is crucial in reducing the risk of hematoma formation [8].

Clinical presentation and challenges

On the 11th postoperative day, our patient presented with severe calf pain and swelling. The affected leg was tense, with shiny skin, and peripheral pulses were absent. These symptoms strongly suggested compartment syndrome. Given the clinical urgency, the patient was immediately taken to the operating room without performing an ultrasound to rule out deep vein thrombosis (DVT), as DVT was not suspected in this case. The diagnostic challenges posed by the formation of a delayed hematoma underscore the importance of maintaining a high level of clinical suspicion and implementing prompt interventional management strategies.

Preventative strategies

To decrease the risk of hematoma formation, several preventative measures should be considered. Optimizing preoperative assessments is crucial, as each patient requires a tailored risk assessment strategy and management plan, particularly for those on anticoagulants or with multiple comorbidities. Careful maintenance of hemostasis during surgery, along with the potential insertion of drains or the use of fenestrated grafts, should be considered to prevent hematoma formation. Additionally, constant vigilance in monitoring high-risk patients for signs and symptoms of complications is essential to prevent the development of severe postoperative complications [9].

Recommendations and implications

This case highlights the critical importance of thorough postoperative care and monitoring, especially for patients with significant comorbidities and those on antithrombotic medication. It is essential to inform the surgical and nursing teams about the potential risk of compartment syndrome due to delayed hematoma formation and to establish early detection and treatment protocols to reduce the likelihood of such complications. Further research is needed to develop standardized management guidelines and identify specific risk factors associated with delayed hematoma formation.

## Conclusions

This case report highlights a rare and significant presentation of compartment syndrome resulting from delayed hematoma following fibular flap harvest for mandibular reconstruction. Early recognition and appropriate management of this complication are crucial to minimizing its severity. Our findings emphasize the importance of thorough preoperative assessments, meticulous surgical techniques, and attentive postoperative care. Future studies should aim to expand the literature on similar surgical scenarios to enhance our understanding and management of delayed hematoma formation.
